# Longitudinal associations between women’s cycle characteristics and sexual motivation using Flo cycle tracking data

**DOI:** 10.1038/s41598-024-60599-1

**Published:** 2024-05-07

**Authors:** Summer Mengelkoch, Katja Cunningham, Jeffrey Gassen, Anna Targonskaya, Liudmila Zhaunova, Rodion Salimgaraev, Sarah E. Hill

**Affiliations:** 1 Laboratory for Stress Assessment and Research, Department of Psychiatry and Biobehavioral Sciences, University of California, Los Angeles, Los Angeles, CA 90095-7076 USA; 2 Department of Psychology, Texas Christian University, Fort Worth, TX 76129 USA; 3 Flo Health, Inc., Wilmington, DE USA

**Keywords:** Psychology, Endocrinology

## Abstract

In the current research, we used data from a sample of 16,327 menstrual cycle tracking app users to examine the association between menstrual cycle characteristics and sexual motivation tracked over 10 months of app use. Guided by past work that finds links between menstrual cycle characteristics related to conception risk and sexual motivation, we found that (a) between-women, shorter (*r* = − 0.04, *p* = 0.007), more regular cycles predicted small increases in sexual motivation (*r* = − 0.04, *p* = 0.001); (b) within-women, shorter cycles predicted greater sexual motivation that month (*r* = − 0.04, *p* < 0.001) and (c) the next month (*β*s: − 0.10 to − 0.06, *p*s < 0.001), but (d) changes in sexual motivation did not reliably precede changes in cycle length (*β*s: − 0.01 to 0.02, *p*s > 0.15). Within-woman analyses also revealed that (e) shorter cycles were followed by more frequent reports of fatigue (*β* = − 0.06, *p* < 0.001), insomnia (*β* = − 0.03, *p* < 0.001), and food cravings (*β* = − 0.04, *p* < 0.001). Together, results suggest that menstrual cycles characteristics and sexual motivation may covary together in ways that reflect changing investments in reproduction. Small effect sizes and lack of experimental control warrant cautious interpretations of results.

## Introduction

Evolutionary researchers have proposed that women’s sexual motivation should be highest at times in the ovulatory cycle when conception risk is high. This relationship is predicted to occur because it would have benefitted women’s reproductive success, by encouraging sexual behavior during the 5–7 days per cycle in which sex could result in pregnancy. Consistent with this hypothesis, several studies find that women’s sexual motivation and behavior each peak near ovulation [e.g.,^1–6^; for reviews, see^7,8^]. This suggests cyclical changes in ovarian hormones related to female fertility may play a mechanistic role in guiding female sexual motivation, with women’s sexual desire and behavior varying as a function of the probability of conception.

Although much work has examined the relationship between conception risk and changes in women’s sexual motivation and behavior occurring within individual women’s cycles, less research has investigated between-woman and between-cycle differences in conception risk and sexual motivation (for an exception, see^2^). As such, scientists know very little about relationships between cycle characteristics and sexual motivation outside of a woman’s individual cycle. Further, because much past research has focused on effects of ovulation (studying cycles during which ovulation has been confirmed or is assumed to have occurred from the results of hormone tests or counting methods), these methods remove a potentially important source of data for testing evolutionarily-derived predictions about women’s sexual psychology: non-conceptive cycles. This is an important source of variability, as there are considerable differences in conception risk occurring between cycles (e.g.,^2,9,10^) and between women^11^.

If natural selection has shaped the human female nervous system to be sensitive to internal cues to conception risk, then in addition to finding that women exhibit greater sexual motivation on days when conception risk is relatively high (e.g.,^1–5^), we should also find that women exhibit greater sexual motivation during cycles that are more likely to be conceptive than during those less likely to be conceptive. Additionally, we should expect women who have more frequently occurring conceptive cycles will exhibit greater overall sexual motivation than women with less-frequently occurring conceptive cycles. Examining the relationship between sexual motivation and conception risk at each the level of the cycle and the woman may provide a valuable complement to existing methods of data collection and analysis for researchers interested in the links between conception likelihood and women’s motivational states, as it has the potential to address some limitations associated with day-of-cycle based methodologies (i.e., inability to account for non-conceptive cycles).

Researchers use cycle characteristics including cycle length (varying within and between women^12^) and cycle length variability (varying between women^13^), which typically co-vary with each other (e.g.,^14^), to compare conception likelihood between-women and between-cycles, as shorter, more regular cycling is associated with higher fecundity. Research finds that women with longer, more irregular cycles take longer to conceive and are more likely to experience infertility compared to women with shorter, more regular cycles^15,16^. Additionally, longer, more variable cycles are also associated with a greater incidence of additional health problems that can interfere with conception, including polycystic ovary syndrome (PCOS;^17^), diabetes^18^, breast cancer^19^, cardiovascular disease^20^, myocardial infarction^21^, and inflammatory bowel disease^22^. Accordingly, if the human female nervous system is indeed sensitive to internal cues to conception likelihood, we should expect to see shorter, more regular cycles characterized by more frequent reporting of sexual motivation than longer, less regular cycles.

## The current research

**Figure 1:**
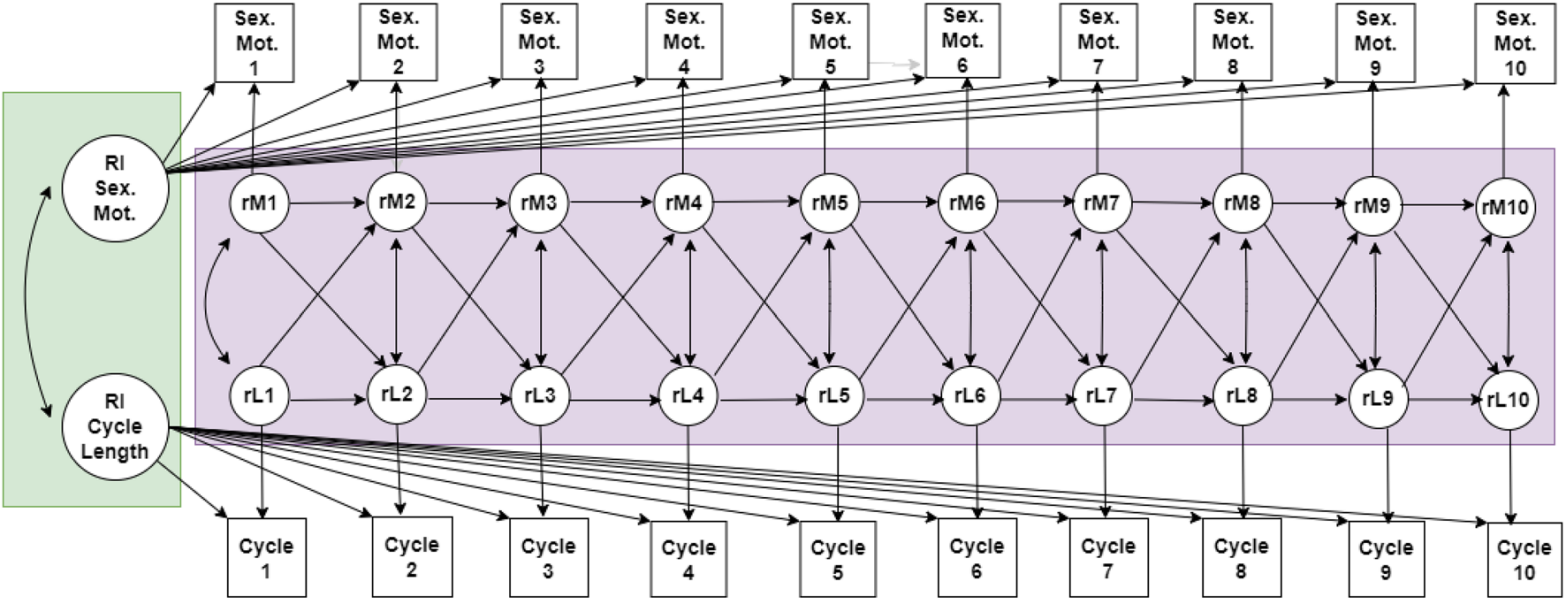
Diagram of final longitudinal model. Covariates at the between level were also tested (see ‘Method’ for full list) but are not shown here. Parameters in green refer to the between-individual portion of the model; parameters in pink refer to the within-individual portion of the model. Squares indicate observed variables (error variances fixed to 0) and circles indicate latent variables. Factor loadings were fixed at 1, per convention. Straight, single-sided arrows refer to structural regression paths, including both the autoregressive and cross-lagged paths. Curved doubled-sided arrows represent covariances between factors. Straight double-sided arrows represent covariance of residuals within each occasion. Cycle variability was included as a between-individual variable measured as the coefficient of variation between all cycles (not pictured here). RI, random intercept; Sex. Mot., sexual motivation; rM, residual for sexual motivation; rL, residual for cycle length.

The current research examined the relationship between women’s menstrual cycle characteristics and sexual motivation using data collected from a large sample of naturally cycling (i.e., free from hormonal contraceptive use) American women (*N* = 16,327) over the course of 10 months. Sexual motivation was operationalized as the number of times women logged sex (protected or unprotected), high sex drive, or masturbation across each cycle. We predicted that (a) at the sample level (i.e., between-women), women with shorter, more regular cycles would report more frequent sexual motivation and behavior than women with longer, more variable cycles and (b) within-women, cycles that are shorter would be characterized by more frequent reporting of sexual motivation and behavior than cycles that are longer. Further, informed by past research findings that exposure to men^23^, sexual contact^24,25^, and mating effort^26^ each predict decreased cycle length in subsequent cycles, we examined whether (c) sexual motivation and behavior in one cycle would predict subsequent cycle length. Finally, because energetic resources within an organism are finite, energetic investments in one domain (i.e., sexual motivation) often come at the cost of energetic investments in other domains (i.e., somatic health), we also explored (d) if higher sexual motivation when cycles were shorter would coincide with the increased reporting of physical complaints, including fatigue, insomnia, and food cravings. This is presented as a supplemental analysis. We estimated a random intercept cross-lagged panel model (RI-CLPM; see Fig. 1) to test our predictions.

The original pre-registration is available here: https://osf.io/4bztn/?view_only=c7dc5278161845e58aab422e6eb36ea9, but see also https://osf.io/t6zuk/ for explanations of all deviations. Please note that in our original pre-registration (titled “Life History Trade-offs and Menstrual Cycle Length and Regularity”), we indicated, “…we predict that women investing more in mating effort will have shorter, more regular cycles. While in our previous work we predicted that changes in mating effort would precede shifts in cycle length, we will test the direction of this relationship in the current project.” Our predictions at the time of the original preregistration lacked specificity, and have since been refined and clarified in the current manuscript.

## Results

All analysis code and output are available on the Open Science Framework (https://osf.io/t6zuk/).

### Model 1—contemporaneous correlations between cycle length, variability, and sexual motivation

Model fit indices revealed good model fit for all models (see Supplementary Materials Table S1 for fit indices and model comparison). Further, consistent with past research (e.g.,^14^), results revealed that women with longer cycles tended to have more variable cycles (i.e., coefficient of variation; *r* = 0.16, *SE* = 0.01, *t* = 13.12, *p* < 0.001). Additionally, longer cycles (*r* = − 0.04, *SE* = 0.01, *t* = − 2.73, *p* = 0.006) and greater variability between cycles (*r* = − 0.04, *SE* = 0.01, *t* = − 3.47, *p* = 0.001) were each related to slightly lower sexual motivation. Complimentary results were found within-women. Women reported lower sexual motivation during longer cycles than they did during shorter cycles (*r* = − 0.04, *SE* = 0.004, *t* = − 9.12, *p* < 0.001). Although effect sizes were small, these results provide preliminary evidence of contemporaneous links between cycle length and sexual motivation.

### Model 2—temporal order of relationships between sexual motivation and cycle length

Full statistics are available in the Supplementary Materials (Tables S2–S4). Model 2 again revealed that women with longer cycles had more variable cycles (*r* = 0.16, *SE* = 0.01, *t* = 13.12, *p* < 0.001). Additionally, between-woman differences in average cycle length (*r* = − 0.04, *SE* = 0.01, *t* = − 2.68, *p* = 0.007), and variability (*r* = − 0.04, *SE* = 0.01, *t* = − 3.46, *p* = 0.001), were again negatively related to between-woman differences in sexual motivation. Within-women, autoregressive relationships revealed that longer cycles were typically followed by shorter cycles (and vice versa) (*βs* = − 0.10 to − 0.04, *p*s < 0.001), but that higher than average sexual motivation during one cycle was typically followed by continued high rates of sexual motivation during the subsequent cycle (*βs* = 0.16–0.59, *p*s < 0.001). Cross-lagged relationships revealed that sexual motivation during a given cycle did not reliably predict cycle length at the next cycle (*βs* = − 0.02 to 0.02, *p*s > 0.14). However, longer cycles were typically followed by reductions in sexual motivation the following cycle (*βs* = − 0.10 to − 0.06, *p*s < 0.001). Together, these results suggest that women with longer, more variable cycles also tend to score lower on measures of sexual motivation. Within-women, longer cycles are typically followed by decreases in sexual motivation, and shorter cycles are typically followed by increases in sexual motivation. While the sizes of the effects of cycle length on sexual motivation were small, they were observed consistently across cycles.

Follow-up models breaking sexual motivation up into its individual components revealed a similar pattern of results. Longer cycles were followed by fewer logs of unprotected sex (*β* = − 0.08, *SE* = 0.004, *t* = − 22.34, *p* < 0.001), protected sex (*β* = − 0.04, *SE* = 0.004, *t* = − 10.72, *p* < 0.001), high sex drive (*β* = − 0.04, *SE* = 0.004, *t* = − 10.73, *p* < 0.001), and masturbation (*β* = − 0.04, *SE* = 0.004, *t* = − 9.25, *p* < 0.001). This latter finding is of interest as previous research has found that cycle variability is linked to sexual activities involving a partner, but not self-stimulation^27^. Contrary to the primary model results, increases in a woman’s number of unprotected sex logs were followed by decreases in subsequent cycle length (*β* = − 0.01, *SE* = 0.004, *t* = − 2.50, *p* = 0.01). Cycle length was not predicted by protected sex (*β* = 0.01, *SE* = 0.004, *t* = 1.75, *p* = 0.08), masturbation (*β* = 0.002, *SE* = 0.004, *t* = 0.56, *p* = 0.58), or sex drive (*β* = 0.01, *SE* = 0.004, *t* = 1.55, *p* = 0.12). As with the model that treated sexual motivation as a composite variable, effect sizes for relationships between cycle length and each sexual log type were small.

### Supplemental analyses

All statistics for supplemental analyses are reported in Supplementary Materials. To explore if cycle length and regularity predicted a trade-off between sexual motivation and physical complaints, such that higher rates of sexual motivation would be accompanied by a greater number of physical complaints, we ran an additional model testing these associations (see Table S5 for effects including physical complaints). Physical complaints included fatigue, insomnia, and food cravings. These analyses revealed that the focal relationships between cycle length and sexual motivation remained unchanged when introducing physical complaints to the model. Further, results indicated that although women with more physical complaints tended to experience less cycle variability (*r* = − 0.06, *SE* = 0.01, *t* = − 5.41, *p* < 0.001), cycle length between women was not related to number of complaints reported (*p* = 0.71). However, within women, longer cycles were followed by fewer logs of fatigue (*β* = − 0.06, *SE* = 0.004, *t* = − 16.15, *p* < 0.001), cravings (*β* = − 0.04, *SE* = 0.004, *t* = − 10.88, *p* < 0.001), and insomnia (*β* = − 0.03, *SE* = 0.004, *t* = − 6.95, *p* < 0.001). While changes in fatigue logs did not predict subsequent cycle length (*β* = 0.01, *SE* = 0.004, *t* = 1.51, *p* = 0.13), increases in both cravings (*β* = 0.02, *SE* = 0.004, *t* = 3.60, *p* < 0.001) and insomnia (*β* = 0.02, *SE* = 0.004, *t* = 4.35, *p* < 0.001) were followed by longer cycles. As with the sexual motivation models, effect sizes for the focal relationships between cycle characteristics and physical complaints were small. However, these supplementary results provide initial support for the hypothesized trade-off between sexual motivation and physical complaints.

### Sensitivity analyses

**Figure 2:**
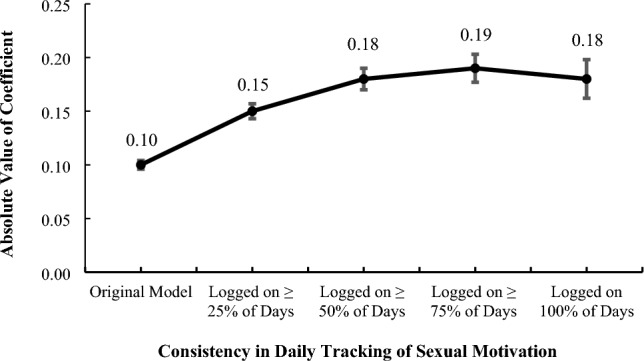
Changes in standardized coefficients (*β*; y-axis) for key effects across sensitivity analyses (x-axis). Shown here are omnibus effects of cycle length on sexual motivation. Error bars reflect standard error of beta coefficients.

We next conducted a sensitivity analysis to ensure that our results could not be attributed to biases emerging from participants’ unreliable tracking of the target variables. Specifically, we examined the extent to which effect sizes changed when we included only women who were consistently logging sexual activity. Full results for the sensitivity analyses are available in the Supplementary Materials (see Table S6). Results revealed that not only did the pattern and significance of the primary results remain constant across the sensitivity analyses, but effect sizes for the focal relationship between cycle length and sexual motivation increased considerably. As is shown in Fig. 2, the standardized coefficient for the overall effect of cycle length on sexual motivation nearly doubled from the original model to the model including only cycles with complete data (i.e., sexual motivation was logged on 100% of cycle days), but was still modest. A final follow-up model revealed that results were also unchanged when women approaching menopause (i.e., > 40-years-old) were excluded from the analysis.

## Discussion

Research finds associations between fertility within the ovulatory cycle and sexual motivation, with women exhibiting greater sexual motivation when conception is possible^2,28^. We examined whether previously unexplored cycle-based characteristics—including cycle length and variability (within- and between-cycle predictors of fertility, respectively)—were related to within- and between-women differences in sexual motivation by testing the following predictions: (a) between-women, women with shorter, more regular cycles would report more frequent sexual motivation and behavior than women with longer, more variable cycles and (b) within-women, cycles that are shorter would be characterized by more frequent reporting of sexual motivation and behavior than cycles that are longer. We also explored the (c) direction of associations between cycle characteristics and sexual motivation, and (d) if higher frequencies of logged sexual motivation in the context of shorter cycles coincided with higher frequencies of logged fatigue, insomnia, and food cravings.

Results largely supported our hypotheses. First, consistent with previous work, we found women with shorter cycles also reported more regular cycles than women with longer cycles (e.g.,^14^) and women with shorter, more regular cycles reported higher sexual motivation compared to women with longer, irregular cycles^26^. Further, within-women, shorter cycles were related to within-woman increases in sexual motivation. Our exploratory prediction that sexual motivation in one cycle would predict length of the following cycle was not supported. While sexual motivation did covary with cycle length within each cycle, sexual motivation in any one cycle did not reliably predict the length of the subsequent cycle. In the current work, we instead found evidence that cycle length predicts subsequent sexual motivation, with increased length of one cycle predicting decreased sexual motivation during the subsequent cycle. The lack of support for this prediction could suggest that links between sexual motivation at time 1 and cycle length at time 2 found in previous research^25^ may have emerged as a byproduct of the strong within-cycle association between sexual motivation and cycle length. Lagged relationships between sexual motivation and length of the next cycle may also have been overshadowed by the tendency for longer cycles to be followed by shorter cycles (and vice versa). This pattern is consistent with the idea of cycle length maintenance effects, similar to what is observed in other complex biological systems^29,30^ and the general statistical tendency of regression to the mean^31^. Whether this pattern represents an internal mechanism which regulates cycle length in women, or if this a simply a statistical artifact, needs to be explored in future research.

While our latent variable of sexual motivation did not reliably predict subsequent cycle length, one of its components—unprotected sex—did. Although the effect size was quite small, we found increases in unprotected sex were followed by decreases in cycle length. This result could indicate that cycle length is sensitive to sexual behavior, when that sexual behavior is more likely to be conceptive (i.e., without condom use, in a manner consistent with sexual behavior throughout our evolutionary history), as similar results were not found for protected sex or masturbation. Future research is needed to understand the mechanistic underpinnings of such links. For example, perhaps the female hypothalamic-pituitary–gonadal (HPG) axis is sensitive to chemosensory elements in male ejaculate that play a role in the regulation of cycle length. As noted, additional research is needed before drawing any conclusions about the temporal relationship between sexual motivation and cycle characteristics and their mechanistic underpinnings, especially given the small effect sizes observed in the current research.

### Associations between cycle characteristics, physical complaints, and sexual motivation: potential evidence of energetic trade-offs

Life history models propose that all organisms must make trade-offs in their energetic investments across the many domains of life^32–34^, as energy spent on reproduction, for example, cannot also be spent combatting disease. Indeed, much research finds evidence of such trade-offs within- and between-organisms, with increased investment in reproductive function often co-occurring with decreases in somatic maintenance and vice versa^11,35^, which are evident when shifts in investment are large (e.g., in seasonally breeding animals)^36,37^. Accordingly, if shorter, more regular cycles are linked to more frequent sexual motivation in women, this sexual motivation likely comes at a cost in other domains. Here, we explored if spending energy on reproductive functions and sexual motivation and behaviors might come at the cost of sleep or diet, two aspects of somatic investment which vary across the menstrual cycle in women^38,39^, by assessing rates of logging fatigue, insomnia, and food cravings. Although these measures are not perfect assessments of somatic investments as a whole, assessing the associations between these physical complaints and both sexual motivation and cycle characteristics allows for an initial exploration of the potential that investment in sexual motivation might come at the cost of investment in somatic health (see Supplemental Materials for more detailed explanations of these predictions and results).

Consistent with predictions drawn from life history trade-off models, we found women reporting higher rates of sexual motivation also reported higher rates of insomnia, fatigue, and food cravings. Although the physical complaints assessed in the current study are relatively minor and heterogeneous (insomnia, fatigue, and food cravings), each was related to sexual motivation in ways predicted by life history trade-off models. Specifically, these results demonstrate that energy expended on sexual motivation might come at the cost of investment in sleep and diet. However, interpretation of these results warrants caution as mechanistic explanations for these relationships were not captured by the Flo tracking app, and fatigue, insomnia, and food cravings are an imperfect proxy for somatic maintenance as a whole. It is possible that these patterns instead are evidence of alternative, unexplored mechanistic processes. Future research should investigate other indicators of potential somatic trade-offs, especially those relating to immune function and inflammatory processes.

Past researchers find that beyond reporting increased sexual desire in the late follicular phase of their cycles (e.g.,^2^), women also report decreased food cravings during this time, and increased food cravings during the luteal phase (e.g.,^38^), when sexual desire is lower. The current results may appear to contradict these findings, as we found that women reported increased food cravings during cycles in which they also reported higher sexual motivation. However, if food cravings and sexual desire are indeed traded-off against one another, one might expect that both would simultaneously occur at high or low rates during any one full cycle. Although it has been documented that physical health concerns are associated with longer, irregular cycles and infertility^16–22^, future work investigating the links between cycle-based characteristics and energetic investment in each mating, somatic maintenance, immune function, and parenting could reveal novel predictors of subfertility in women, as well as novel intervention strategies to promote fertility.

### Strengths and limitations

Among the strengths of the current research are that we analyzed a robust dataset from a large, diverse sample of women who were followed longitudinally over 10 full cycles. Although these women were not randomly sampled from the U.S. population, the size and diversity of the sample provides some confidence in the generalizability of the findings. Further, participants logged data for their own benefit, decreasing concerns of inaccuracy in women’s self-reports. Finally, the target analyses were highly powered, allowing us to test complex and alternate models.

Despite these strengths, the current research has some notable limitations. In the current study, the cycle was the smallest unit of analysis, preventing within-cycle analyses investigating mechanistic relationships between cycle characteristics and sexual motivation. Although using a longitudinal design allowed us to investigate temporal changes in these relationships, we cannot make causal claims about the impact of sexual motivation (or physical complaints) on cycle characteristics. Such research would likely require experimental animal models or quasi-experimental studies whereby cycle characteristics of women taking medications which exaggerate trade-offs between reproductive and somatic investment (e.g., fertility treatment, immunosuppressants) are compared to those of unmedicated women.

An additional limitation of the current work is its reliance on specific data collected by the mobile health application. There are many unmeasured variables we cannot account for in our analyses, such as cycle phase, conception risk on a given day, and number of children (i.e., mating success) which may impact cycle characteristics (e.g.,^40^). Further, cycle characteristics are impacted by the occurrence of non-ovulatory cycles, conceptions, and spontaneous abortions, which we were unable to account for in our analyses. For instance, it is possible that spontaneous abortion following conceptive sexual behavior could result in a longer subsequent cycle length and increased cycle variability. Such an effect would represent one potential mechanism driving the association between longer cycles and less regular cycles in some women that we were unable to test directly. While this could have occurred in some women in the current study, the results suggest that the effects of spontaneously aborted pregnancy following unprotected sex did not occur often enough to substantively drive the results in our sample, as we found that unprotected sex acts were followed by shorter, as opposed to longer cycles. Further, if unprotected sex was meaningfully increasing cycle length sporadically (and in turn, increasing cycle variability) due to spontaneous abortion, we would expect to see that unprotected sex predicted longer and less regular cycles compared to protected sex, which was also not the case. Accounting for events such as spontaneously aborted pregnancies (if they occurred) through strict methodological or statistical control could increase the precision of our explanations for the associations reported. Other unassessed factors, such as some cycles containing more weekend days than others, when women are found to engage in heightened sexual behavior^5^, could also impact patterns of sexual motivation. Moreover, women may have different intensity thresholds for reporting variables (i.e., high sex drive, fatigue), potentially introducing systematic error.

Although Flo was marketed as a cycle tracking application at the time these data were collected, we do not know if women were using the app to help them avoid conception, conceive, or track their periods. More information about women’s fertility intentions and use of contraception would provide additional context to the patterns reported here. In the current study, no women were using hormonal contraception, and we assessed the use of protection during sex at the level of each sex act, as opposed to at the level of each woman, as many women choose to use or not use protection differently in different contexts, at different times, or with different partners. However, we did not assess if women had a tubal ligation, had been tested for infertility, or how each woman defined protected versus unprotected sex. It is possible that women with tubal ligations were having unprotected but non-conceptive sex, and as such, not all unprotected sex acts in the current study should be assumed to be potentially conceptive. As no barrier method provides perfect protection, not all protected sex acts should be assumed to be non-conceptive. Further, while protected sex typically means a barrier method was used, some women may consider withdrawal methods as protected sex. Deeper information about women’s contraceptive use and fertility intentions should be collected in future research.

There was also substantial variation in the frequency that women logged the variables we analyzed. We attempted to account for individual differences in logging activity both by focusing on within-women relationships and performing sensitivity analyses of women with different logging frequencies. Although the results of our sensitivity analyses revealed that effect sizes for the focal relationships between cycle length and sexual motivation grew considerably larger when we limited the analyses only to women who consistently tracked these variables, the influence of heterogeneity in logging frequency on the present results cannot be ruled out. Furthermore, effect sizes for relationships between cycle characteristics and logging activity in the primary models were generally small. Small effect sizes in this context could be attributable to the inherent noisiness of cycle length data or inconsistency in behavioral logs (as is suggested by the sensitivity analyses), but may also indicate that even if consistent, relationships between cycle characteristics and sexual behavior are weak. Non-random missingness of data could also introduce bias to effect size estimation in one direction or the other.

Due to these limitations of the data, the practical significance of relationships found in the current research is unknown. Therefore, caution should be exercised when interpreting the results, and additional research is needed to determine any potential practical or clinical implications of these findings. For comparison, though, one previous study examining the effect of cycle frequency on sociosexual orientation found a standardized beta of 0.38^25^, which is substantially larger than our own. However, that study collected a relatively small sample size (*n* = 176), was cross-sectional, women self-reported their average cycle length, and the study only involved college students. Another study that investigated the effects of cycle-related hormonal milieu on sexual frequency—which we expect to play a role in our pattern of results—found the within-woman effects (*β*) of estradiol and progesterone ranged from 0.06 (progesterone) to 0.22 (estradiol)^6^. While modest, the effect size for estradiol resembles that of cycle length in our sensitivity analysis that removed all cycles with incomplete sexual log data (*β* = 0.18). Regarding the impact of conception likelihood on sexual desire, more generally, one study found relatively small standardized coefficients ranging from -0.04 (single women) to 0.20 (women in relationships)^41^, while another found modest Cohen’s *d* values (0.21–0.25)^42^. The latter study, however, did find larger effect sizes for ovulatory shifts in frequency of masturbation (*d*: 0.37–0.45) and sexual fantasies (*d*: 0.47–0.76). These studies offer a more fine-grained analysis of relationships between cycle characteristics and sexual motivation, but even still, effect sizes were typically small. Future research should ensure adequate power to detect effects of this magnitude, use clinically validated instruments to measure key variables, and utilize more rigorous methodological control than is possible when using app data, in order to maximize the clinical relevance of future work investigating associations between cycle characteristics and sexual motivation.

Finally, because we relied upon data being collected by the mobile health application, we did not examine the biological mechanisms that mediate relationships between menstrual cycle characteristics, sexual motivation, and physical complaints. Based on associations observed in past research between shorter cycle lengths and elevated estradiol levels^43^ and between elevated estradiol levels and heightened sexual desire^2,3^, it is likely that hormonal signals are driving the relationships found in the current study. Although we suspect that the hormonal signals that regulate cycle length (e.g.,^44^) also impact cycle-to-cycle rates of sexual desire by providing cues of a given cycle’s conceptive likelihood, this relationship could not be established in the current work. As menstrual cycles are impacted by perceived stress and the activities of the hypothalamic–pituitary–adrenal axis^9^, sex steroid hormones^45^, and other endocrine and immune signals (e.g., cytokines;^46^), further research is needed to explore these potential biological mechanisms which likely impact the associations reported in the current work. One possibility is that, beyond providing a cue to a woman’s fertility, the length of a woman’s cycle might also serve as an indicator of her future fecundity. A recent meta-analysis revealed that women with shorter cycle lengths (i.e., cycle lengths between 21 and 27 days), exhibited reduced ovarian reserves compared to women with normal (i.e., 28–31 days) or longer (i.e., 32–35 days) cycle lengths^47^. Given that women may have a finite number of ovarian follicles, it could be that shorter cycle lengths provide a cue to the body to prioritize current reproduction, not only because a woman’s current fertility is high, but also because her ability to conceive in the future might be limited, although this explanation is speculative. Continuing collaboration between women’s health tracking applications and researchers may be a promising avenue to answer these and other related questions.

In sum, the current results provide evidence that menstrual cycle length and regularity are related to sexual motivation. Shorter, more regular cycles are associated with higher rates of sexual motivation. Further, higher rates of sexual motivation coincided with increased reporting of fatigue, insomnia, and food cravings, suggesting that increased sexual motivation may come at a cost to one’s somatic health, potentially impacting sleep and diet, although this should be explored in future research. Focal effect sizes for relationships between cycle characteristics and sexual motivation in the current study were very small (e.g., *β*s: − 0.10 to − 0.06), however, that effect sizes became larger when reducing noise (sensitivity analysis: *β*s: − 0.19 to − 0.15), suggests that future research using tightly controlled research methods is needed to boost precision of these estimates. Together, these results suggest that while there is substantial variability in women’s cycle characteristics, differences in cycle length both within- and between-women are not merely random. Instead, they may reflect coordinated shifts in response to internal and environmental cues to the costs and benefits of reproductive investment. The current work sets the stage for future studies to examine the influence of health, lifestyles, and social environments on menstrual cycle dynamics.

## Method

### Participants

Participants included 16,327 naturally cycling (i.e., free from hormonal contraceptive use) users of the Flo Period and Health Tracking mobile application from the United States of America. Women’s ages ranged from 18 to 57 (*M*_age_ = 27.19, *SD*_age_ = 7.49). All data were de-identified and aggregated prior to transfer from Flo Health Inc. Additional characteristics of the sample are displayed in Table 1.

**Table 1:** Demographic characteristics of the sample.

Characteristics of the sample (N = 16,327)
Age (18–57): *M* = 27.19, *SD* = 7.49
Race/Ethnicity
White: 45.45% (*n* = 7420)
African American: 15.05% (*n* = 2458)
Asian: 4.46% (*n* = 729)
Hispanic: 18.99% (*n* = 3100)
Indian: 0.86% (*n* = 141)
Other: 3.61% (*n* = 589)
Did not respond: 11.58% (*n* = 1890)
Relationship status
No relationship: 18.38% (*n* = 3000)
Unstable relationship: 7.92% (*n* = 1293)
Stable relationship: 35.78% (*n* = 5842)
Married: 23.21% (*n* = 3790)
Did not respond: 14.71% (*n* = 2402)
Smoking
Yes: 13.72% (*n* = 2240)
No: 86.28% (*n* = 14,087)
Chronic Illness/Disease
Yes: 7.82% (*n* = 1276)
No: 92.18% (*n* = 15,051)
Weight (lbs.): *M* = 158.64, *SD* = 48.16

### Procedure and materials

Flo is a health and period tracking mobile application that allows users to enter information about health, pregnancy status, and menstrual cycle characteristics, and offers ovulation and pregnancy calendars (for more information, see https://flo.health/). Scientists at Flo aggregated and de-identified data from 20,000 users in the United States as part of a collaboration agreement with the authors. (Note: While the authors sought to analyze as much data as possible, Flo offered data for one year from 20,000 women that fit the study inclusion criteria, a large proportion of their userbase at the time, with a restricted set of variables. Sample size determinations balanced data security concerns with power demands.) Of these users, 3673 were under the age of 18 and therefore excluded from data analysis, yielding a final data analytic sample of 16,327 women. All users in the study provided informed consent for the use of their de-identified and aggregated data for research purposes by accepting the Privacy Policy during registration. This research was exempt from Texas Christian University’s institutional board review as data were de-identified and aggregated prior to analysis, and all research was performed in accordance with the declaration of Helsinki.

**Figure 3:**
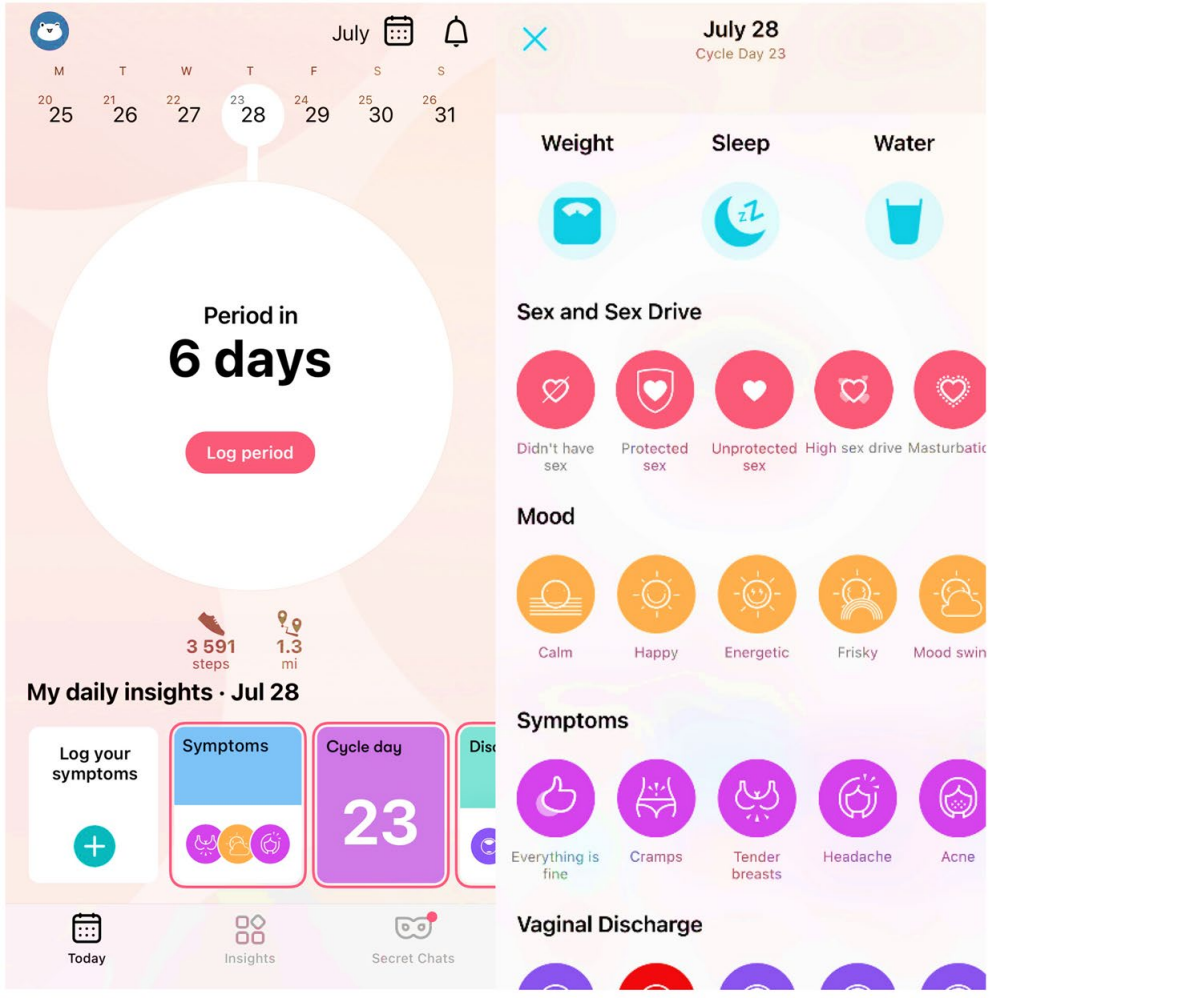
Flo period and health tracking mobile application interface. Application users have the option to log daily symptoms from the application’s home screen (left). After choosing to log symptoms, users are presented with a variety of experiences and symptoms to report each day (right).

Data included input from the onboarding survey, as well as measures collected for 12 consecutive menstrual cycles, collected in 2018–2019. Single timepoint onboarding data included age (continuous), weight (continuous), relationship status (0 = single, 1 = unstable relationship, 2 = stable relationship, 3 = married), smoking status (0 = no, 1 = yes), whether or not the user reported a chronic illness or disease (0 = no, 1 = yes), and race/ethnicity (dummy-coded). Longitudinal data included cycle length, along with the number of times that sex (protected or unprotected), high sex drive, or masturbation were logged across each cycle, as well as the number of times fatigue, insomnia, or food cravings were logged across each cycle for 12 consecutive cycles. Women self-reported these measures by logging their occurrence throughout their cycles (see Fig. 3 for symptom logging interface).

The primary purpose of the current project was to examine longitudinal relationships between sexual motivation and cycle characteristics. To this end, we combined the number of logs each cycle for protected sex, unprotected sex, high sex drive, and masturbation into a sum composite representing sexual motivation. Measures included in the current study were selected from those already being collected within the Flo application. We included all measures of sexual motivation/behavior which were available. Follow-up models examined unique effects for each individual log type. Descriptive statistics for these measures, as well as cycle length are displayed in Table 2. We also included a measure of each woman’s cycle variability by calculating the coefficient of variation for all cycles included in the analyses (i.e., ratio of standard deviation to the mean).

**Table 2:** Descriptive statistics for key study variables.

Descriptive statistics, cycles 2–10	
Variable (min–max)	*M (SD)*
Average cycle length (17–41.29)	29.07 (2.78)
Total number of logs	
Protected sex (0–151)	5.26 (12.51)
Unprotected sex (0–193)	14.53 (23.56)
High sex drive (0–267)	6.50 (16.13)
Masturbation (0–233)	4.83 (12.33)
Fatigue (0–336)	13.22 (25.04)
Insomnia (0–291)	3.07 (9.51)
Food cravings (0–214)	6.10 (12.94)
*Note*. Total number of logs is the sum across cycles 2–10.	

### Data analysis plan

All analysis code, including output, is available on the Open Science Framework (https://osf.io/t6zuk/). Data were collected over the course of 12 menstrual cycles. Because some women began and ended the study at different points in their cycles, data from the first and final cycles were incomplete (i.e., cycle lengths were cut off depending on start and/or end dates, see Supplementary Materials Fig. S1). These data were therefore excluded, leaving 10 cycles of full data for each user for a total of 163,270 cycles. Guided by the results of recent research tracking more than 600,000 cycles^48^ (see also^49,50^), cycles lasting fewer than 15 days and greater than 50 days were eliminated since 99.9% of all cycles fall within this range. Per convention in cycle tracking data analysis, missing data were excluded pairwise (i.e., only single data points removed, not all data for participant), as these values could be attributable to user error (e.g., forgetting to log a period). After this exclusion, data for 7.75% of women were missing for at least one cycle; missing data were handled using maximum likelihood estimation^51,52^.

One limitation of cycle-tracking data is that the number of days available to log data in a given cycle is necessarily confounded with cycle length. Women with longer cycles have more days to log events than do women with shorter cycles. We adjusted for this limitation in two ways. First, to examine within-participant longitudinal relationships between time-varying measures (i.e., cycle length, sexual motivation,), we estimated a random intercept cross-lagged panel model (RI-CLPM; see Fig. 1 for model diagram) that modeled covariance between the length of a cycle and the number of logs during that cycle^53,54^. Second, we divided the number of logs of each category by the length of the concurrent cycle (i.e., number of days), thus calculating a “logs per day” rate variable for each log type (see below for more information).

The RI-CLPM is a recently developed alternative to the traditional CLPM that provides unique solutions to several statistical issues associated with the latter model^53–56^. In particular, the traditional CLPM assumes that individuals’ values for a given variable fluctuate around the overall sample mean over time meaning that (a) no stable between-individual differences in this variable across measurements are modeled (i.e., the mean of all time points is assumed to be equal for each person) and thus (b) within-individual (temporal differences) and between-individual (stable, trait) effects are conflated in the estimation of autoregressive and cross-lagged parameters^53–56^.

In contrast, the RI-CLPM includes a random intercept in the model that captures stable between-person differences (between-individual portion of model). Specifically, the intercept represents each individual’s time invariant deviation from the grand mean. The within-individual portion of the model consists of latent variables representing the difference between a variable’s observed value at each time point and its expected value based on the grand mean and random intercept. As a result, autoregressive and cross-lagged effects specified between these latent variables exclusively describe relationships at the individual level across occasions^53–56^. For the current research, the RI-CLPM allowed us to examine predictors (e.g., age, weight, relationship status) of between-participant differences in cycle length, variability, sexual motivation, and physical complaints, as well as test whether within-individual, timepoint-based differences in sexual motivation and physical complaints were related to cycle length while controlling for autoregressive effects (e.g., sexual motivation itself at one time point predicting sexual motivation at the next time point).

Regarding point *a* above, women with longer cycles had a greater number of days to log events. Covariance between total number of logs and cycle length is modeled in the within-individual portion of the RI-CLPM. However, the confounding of cycle length and number of days to log events may bias estimation of relationships between variables measured between participants (e.g., age, weight, relationship status) and the intercept (representing stable between-person differences) of measures varying across cycles (i.e., sexual motivation). Accordingly, to test between-participant relationships, the number of each log type was divided by the length of the concurrent cycle, creating “rate” variables representing logs per day within a cycle. Importantly, due to the control provided in the within-women portion of the model, and our use of rate variables in the between-women potion of the model, we can be fairly confident that patterns found both within- and between-women do not emerge in response to between-user differences in logging frequency.

Models were tested iteratively, and significant covariates were controlled for in all models. First, as an initial, proof-of-concept model, we tested contemporaneous correlations between cycle length and sexual motivation to examine whether these variables are related across time (Model 1). For this initial model, within-individual relationships were constrained as equal over time as an omnibus test of links between sexual motivation and cycle lengths (all paths were freed in subsequent models). Next, to examine the temporal order of associations between cycle length and sexual motivation, cross-lagged paths were introduced in the model such that we tested whether changes in sexual motivation preceded changes in cycle length (or vice versa) (Model 2; see Fig. 1 for diagram). Follow-up models were conducted for each individual sexual log type to examine whether results for the composite measure were consistent across component variables (i.e., unprotected sex, protected sex, masturbation, sex drive).

To examine longitudinal relationships between sexual motivation, physical complaints, and cycle characteristics per our secondary hypothesis (Model 3, see Supplementary Materials), we analyzed the number of logs each month for fatigue, insomnia, and cravings as individual variables, as well as a sum representing overall physical complaints. To streamline reporting of individual log models, equality constraints were added to regression paths in order to produce an omnibus test for each effect over time.

After estimating the final model, we tested a number of equality constraints to examine whether more parsimonious models provided a better fit to the data than the freely estimated model (i.e., without constraints). These included (a) constraining the covariances between variables at each time point to be equal, (b) constraining the autoregressive paths to be equal, and (c) constraining the cross-lagged paths to be equal. These alternative nested models were compared to the unconstrained model using *χ*^2^ difference tests^57^. Because the model without constraints provided the best fit to the data (see Supplementary Materials Table S1 for model fit indices), we report it below as the primary model. Good model fit was also determined by a comparative fit index (CFI) value > 0.95, a root mean square error of approximation (RMSEA) value < 0.05, and a standardized root mean square residual (SRMR) statistic < 0.05.

We also tested an additional follow-up model to examine whether the pattern of results found in the primary model changed when we excluded women over the age of 40. We sought to test the model excluding women over the age of 40 because previous research has found that there is a marked increase in cycle variability during the perimenopausal transition^58^. Results of these analyses are referred to in the main text; statistics are found in the Supplementary Materials.

As a final control for the possibility that unreliable tracking of sexual motivation between- or within-women biased the data in favor of the results found in the primary model, we conducted a series of sensitivity analyses. Specifically, we tested whether the direction or size of the effects found in the primary model changed when restricting analyses only to women who reached certain thresholds of tracking consistency. This is critical because the absence of a log could either mean that the behavior did not occur or that it did occur and was not logged. For sexual motivation, although women were given the option to log “no sex,” many did not use this option at all during the study period (19.6%). It is possible that gaps in reporting could produce spurious relationships between the key variables of interest. The follow-up sensitivity analyses were designed to address this issue by assessing whether the primary results were robust across different levels of certainty in how consistently women were tracking and reporting sexual activity. If the effects decrease in size or change signs when analyses are restricted to only women reliably tracking sexual motivation, serious doubts should be raised about the validity of the main findings. If, on the other hand, effect sizes do not change or become larger as tracking consistency increases, this would suggest gaps in reporting merely add noise to the data and that it is unlikely the previous findings are attributable to bias introduced by unreliable logging activity.

To perform the sensitivity analyses, we first divided the number of logs of “no sex” or sexual activity (i.e., unprotected sex, protected sex, masturbation, or high sex drive) reported each cycle by the number of days in that cycle to determine the percentage of days that sexual activity (or lack thereof) was logged. For example, when a woman logged either some type of sexual activity or “no sex” on each day in the cycle, that cycle would have complete data for sexual motivation. We tested the final model (Model 2) again while restricting the analyses to only cycles during which either no sex or sexual activity was logged on at least (a) 25% of days (40.0% of cycles), (b) 50% of days (20.6% of cycles), (c) 75% of days (13.8% of cycles), and (d) 100% of days (6.3% of cycles).

### Supplementary Information

Supplementary Information.

## Data Availability

Data are not publicly available, but may be made available upon reasonable request and approval from Flo. See https://osf.io/t6zuk/ for code and output files. Preregistration available at: https://osf.io/4bztn/?view_only=c7dc5278161845e58aab422e6eb36ea9.
