# Supplementary material for: Longitudinal associations between women’s cycle characteristics and sexual motivation using Flo cycle tracking data
Source: Sci Rep. 2024 May 7;14:10513. doi: 10.1038/s41598-024-60599-1 (PMC11076276; doi:10.1038/s41598-024-60599-1)
Supplement: Supplementary file 1 — Supplementary Information. [file 41598_2024_60599_MOESM1_ESM.docx]

**Supplemental Materials for:**

Longitudinal associations between women’s cycle characteristics and sexual motivation using Flo™ cycle tracking data

*Summer Mengelkoch^1^, Katja Cunningham^2^, Jeffrey Gassen^1^, Anna Targonskaya^3^, Liudmila Zhaunova^3^, Rodion Salimgaraev^3^ & Sarah E. Hill^2^

^1^ University of California, Los Angeles, Department of Psychiatry and Biobehavioral Sciences, CA, USA

^2^ Texas Christian University, Department of Psychology, Fort Worth TX 76129

^3^ Flo Health, Inc., Wilmington, Delaware, USA

**Declarations of interest**: AT, LZ, and RS are/were employees of Flo Health, Inc. They had no role in the formation of the research hypothesis, nor in the analysis of data.

**Correspondence to:** Summer Mengelkoch, PhD; Laboratory for Stress Assessment and Research, University of California, Los Angeles, California 90095-7076, USA. Email: [smengelkoch@mednet.ucla.edu](mailto:smengelkoch@mednet.ucla.edu)

**Supplemental Research Question**

Beyond research questions examined in the main text, we also explored potential trade-offs between high sexual motivation and reported physical complaints, which included the number of times women logged fatigue, insomnia, or food cravings across each cycle. Because energetic resources are finite, organisms must make trade-offs in their energetic investments across the many domains of life [1-2]. Energy spent on reproduction, for example, cannot also be spent combatting disease. Indeed, much research finds evidence of such trade-offs within- and between-organisms, with increased investment in reproductive function often co-occurring with decreases in somatic maintenance and vice versa [3], which are evident when shifts in investment are large (e.g., in seasonally breeding animals) [4-6]. Accordingly, if shorter, more regular cycles are linked to more frequent sexual motivation, this sexual motivation must come at a cost. In women, spending energy on reproductive functions and sexual motivation and behaviors might come at the cost of sleep, diet, physical activity, immune function, or other somatic investments. Due to the pleiotropic functions of sex in human relationships and limitations of our data, we expect to observe small effect sizes of associations between cycle characteristics, sexual motivation, and physical complaints that could result from decreased somatic investment compared to what would be observed in seasonally breeding animals. Here, we expect that cycles characterized by high rates of sexual motivation will also be characterized by high rates of fatigue, insomnia, and food cravings. Past research finds that within-women, trade-offs have been observed between energetic stress (e.g., food scarcity, sleep deprivation) and reproductive hormone levels (which in turn, impact fertility; [7]). Additionally, past work finds that insomnia, fatigue, and food cravings vary cyclically across women’s cycles [8, 9], indicating that sleep and diet may be sensitive to changes in the cyclical sex steroid hormone levels which also influence sexual motivation. As such, we explored the possibility that (d) increased sexual motivation would come at the cost of somatic investment (here, proxied by increased reporting of insomnia, fatigue, and food cravings).

To examine longitudinal relationships between sexual motivation, physical complaints, and cycle characteristics, we combined the number of logs each cycle for protected sex, unprotected sex, high sex drive, and masturbation into a sum composite representing sexual motivation. We next combined the number of logs each cycle for fatigue, insomnia, and food cravings into a sum composite representing physical complaints. We also analyzed the number of logs each month for fatigue, insomnia, and cravings as individual variables. Measures included in the current study were selected from those already being collected within the Flo™ application. We included all measures of sexual motivation/behavior and physical complaints which were available.

**Supplemental Results**

**Model 3 – Sexual Motivation, Physical Complaints, and Cycle Characteristics**

In a secondary model, we sought to explore if cycle length and variability predicted a trade-off between sexual motivation and somatic complaints, such that higher rates of sexual motivation would be accompanied by a greater number of somatic complaints. Full statistics are displayed in Tables S2–S5. Between-women, results revealed the random intercepts of sexual motivation and physical complaints were positively correlated (*r* = .38, *SE* = .01, *t* = 37.55, *p* < .001), indicating that women with higher sexual motivation also logged more physical complaints. As in Models 1–2, the random intercept of cycle length was negatively correlated with the random intercept of sexual motivation (*r* = -.04, *SE* = .01, *t* = -2.65, *p* = .008), but not that of physical complaints (*r* = -.01, *SE* = .01, *t* = -.37, *p* = .71; physical complaints not tested in Models 1–2). That is, women with longer cycles logged fewer mating-related activities than women with shorter cycles, but logged similar numbers of physical complaints. Again, women with longer cycles also had more variable cycles (*r* = .16, *SE* = .01, *t* = 13.14, *p* < .001). Cycle variability was negatively correlated with the intercepts of both sexual motivation (*r* = -.04, *SE* = .01, *t* = -3.49, *p* < .001) and physical complaints (*r* = -.06, *SE* = .01, *t* = -5.41, *p* < .001), indicating that women with more variable cycle lengths logged fewer sexual activities and physical complaints than women with more regular cycles. Together, these results suggest women with shorter, more regular cycles engaged in more mating-related activity than those with longer, irregular cycles. Further, women with more regular (but not necessarily shorter) cycles, also reported more physical complaints than those with more variable cycles.

Certain covariates also predicted stable, between-women differences in cycle characteristics, sexual motivation, and physical complaints (see Supplementary Materials Tables S2–S5). Women with longer cycles tended to be younger (*p* < .001), in more stable relationships (*p* < .001), and non-smokers (*p* < .001). Greater cycle variability was associated with older age (*p* = .02), and chronic illness (*p* = .046). Furthermore, women reporting greater sexual motivation were younger (*p* < .001), in more stable relationships (*p* < .001), more likely to smoke (*p* < .001), and had higher body weight (*p* = .003) compared to women with less sexual motivation. Lastly, women with more physical complaints were younger (*p* = .04), more likely to report chronic illness (*p* < .001), and in less stable romantic relationships (*p* = .03) than women with fewer complaints.

Within-women, consistent with Model 2, longer cycle length during one cycle predicted a shorter subsequent cycle (all *p*s < .001). Contrary to our predictions about the directionality of relationships between the target variables, fluctuations in sexual motivation and physical complaints did not reliably predict subsequent cycle length. Overall, even when including autoregressive parameters, the model accounted for a very small amount of variance in cycle length across time (*R*^2^ = .002–.01).

The autoregressive parameters for sexual motivation and physical complaints displayed the opposite pattern as those for cycle length. Specifically, increases in sexual motivation and physical complaints during one cycle predicted further increases during the following cycle (all

*ps* < .001). Moreover, longer cycles predicted decreases in both sexual motivation and physical complaints during subsequent cycles (all *ps* < .001), consistent with the hypothesis that cycle length is associated with trade-offs between sexual and somatic investment. Importantly, this result within-women provides evidence that the between-user patterns did not emerge in response to between-user differences in logging frequency.

Follow-up models for individual physical complaints (i.e., breaking physical complaints up into its individual components) logs revealed a similar pattern. Longer cycles were followed by fewer logs of fatigue (*β =* -.06, *SE* = .004, *t* = -16.15, *p* < .001), cravings (*β =* -.04, *SE* = .004, *t* = -10.88, *p* < .001), and insomnia (*β =* -.03, *SE* = .004, *t* = -6.95, *p* < .001). Increases in fatigue logs did not predict subsequent cycle length (*β =* .01, *SE* = .004, *t* = 1.51, *p* = .13). However, increases in both cravings (*β =* .02, *SE* = .004, *t* = 3.60, *p* < .001) and insomnia (*β =* .02, *SE* = .004, *t* = 4.35, *p* < .001) were followed by longer cycles.

**Results of Model Excluding Women over 40 Years Old**

Excluding women over 40 years old did not change the pattern or significance of the results reported in the main text. Specifically, the random intercepts of sexual motivation and cycle length remained negatively correlated (*r* = -.04, *SE* = .01, *t* = -2.66, *p* = .008). The intercept of sexual motivation also remained negatively correlated with cycle variability (coefficient of variation: *r* = -.03, *SE* = .01, *t* = -2.74, *p* = .006).

Regarding the within-person portion of the model, the autoregressive effects for sexual motivation were unchanged by excluding older women. Higher sexual motivation during one cycle continued to predict higher sexual motivation at the subsequent cycle (*β*s: .16–.61, *p*s < .001). The cross-lagged effects of sexual motivation on cycle length were all non-significant (*β*s: -.004–.02, *p*s: .09–.98). The cross-lagged effects of cycle length on sexual motivation, on the other hand, remained significant (*β*s: -.11 to -.06, *p*s < .001).

**Supplemental Discussion**

**Discussion of Individual Differences in Cycle Length and Variability**

Despite a strong relationship between cycle length and cycle regularity across our sample, we found some individual differences in each of these characteristics. Women with more variable cycles tended to be older and more likely to report a chronic illness than those with less variable cycles. This is consistent with previous research linking age and poor health with increased cycle variability [10, 11]. Further, women with longer cycles tended to be younger, in more committed relationships, and were less likely to smoke compared to women with shorter cycles. This could reflect younger women prioritizing career goals over mating success, although future research would be needed to support this notion. While one might not expect women with longer cycles to be more likely to be non-smokers than women with shorter cycles, given associations between cycle length and health, life history models highlight that those with increased sexual motivation are also more likely to engage in behaviors, such as smoking, which infer small costs in the present, but larger costs in the future (e.g., [12]).

References

1. Kaplan, H. S., & Gangestad, S. W. (2005). Life history theory and evolutionary psychology. In D. M. Buss (Ed.), *The Handbook of Evolutionary Psychology* (pp. 68–95). John Wiley & Sons, Inc..
2. Stearns, S. C. (1992). *The evolution of life histories* (Vol. 249, p. xii). Oxford: Oxford university press.
3. Ellison, P. (2003). Energetics and reproductive effort. *American Journal of Human Biology*, *15*(3), 342-351. doi: 10.1002/ajhb.10152
4. Del Giudice, M., Gangestad, S., & Kaplan, H. (2015). Life history theory and evolutionary psychology. *The Handbook of Evolutionary Psychology*, 1-27. doi: 10.1002/9781119125563.evpsych102
5. Ots, I., & Horak, P. (1996). Great tits Parus major trade health for reproduction. *Proceedings of the Royal Society of London. Series B: Biological Sciences*, *263*(1376), 1443-1447. doi: [10.1098/rspb.1996.0210](https://doi.org/10.1098/rspb.1996.0210)
6. Rubach, K., Wu, M., Abebe, A., Dobson, F. S., Murie, J. O., & Viblanc, V. A. (2016). Testing the reproductive and somatic trade‐off in female Columbian ground squirrels. *Ecology and Evolution*, *6*(21), 7586-7595. doi: [10.1002/ece3.2215](https://doi.org/10.1002%2Fece3.2215)
7. Jasienska, G., Bribiescas, R. G., Furberg, A. S., Helle, S., & Núñez-de la Mora, A. (2017). Human reproduction and health: an evolutionary perspective. *The Lancet*, *390*(10093), 510-520. doi: [10.1016/S0140-6736(17)30573-1](https://doi.org/10.1016/s0140-6736(17)30573-1)
8. Gorczyca, A. M., Sjaarda, L. A., Mitchell, E. M., Perkins, N. J., Schliep, K. C., Wactawski-Wende, J., & Mumford, S. L. (2016). Changes in macronutrient, micronutrient, and food group intakes throughout the menstrual cycle in healthy, premenopausal women. *European Journal of Nutrition*, *55*(3), 1181-1188. doi: [10.1007/s00394-015-0931-0](https://doi.org/10.1007/s00394-015-0931-0)
9. Pengo, M. F., Won, C. H., & Bourjeily, G. (2018). Sleep in women across the life span. *Chest*, *154*(1), 196-206. doi: [10.1016/j.chest.2018.04.005](https://doi.org/10.1016/j.chest.2018.04.005)
10. Chiazze, L., Brayer, F. T., Macisco, J. J., Parker, M. P., & Duffy, B. J. (1968). The length and variability of the human menstrual cycle. *Jama*, *203*(6), 377-380.
11. Wolff, J. L., Starfield, B., & Anderson, G. (2002). Prevalence, expenditures, and complications of multiple chronic conditions in the elderly. *Archives of Internal Medicine*, *162*(20), 2269-2276.
12. Griskevicius, V., Tybur, J. M., Delton, A. W., & Robertson, T. E. (2011). The influence of mortality and socioeconomic status on risk and delayed rewards: a life history theory approach. *Journal of Personality and Social Psychology*, *100*(6), 1015.

Table S1

*Model Fit Indices*

| **Model** | ***χ2 (df*)** | ***χ*^2^ Difference** | **CFI** | **RMSEA** | **SRMR** |
| --- | --- | --- | --- | --- | --- |
| Initial Model | 2500.99 (370)*** | - | .98 | .02 | .02 |
| Final Model with No Constraints | 2602.97 (344)*** | - | .97 | .03 | .02 |
| Final Model with Covariances Constrained | 2624.53 (352)*** | 21.56*** | .97 | .03 | .02 |
| Final Model with Autoregressive Constraints | 3628.03 (360)*** | 1025.06*** | .96 | .03 | .02 |
| Final Model with Cross-Lagged Constraints | 2658.19 (360)*** | 55.22*** | .97 | .03 | .02 |

*Note.* CFI = comparative fit index, RMSEA = root mean square error of approximation, SRMR = standardized root mean square residual.


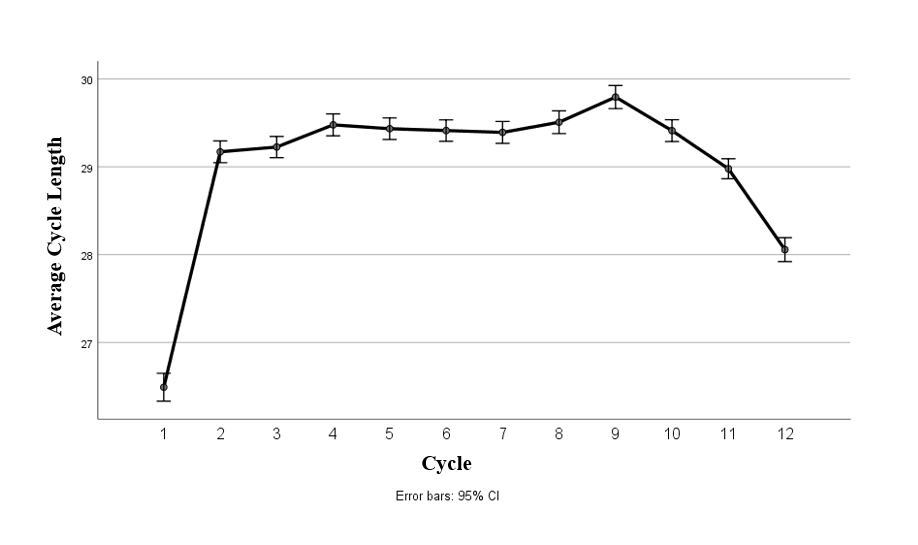


*Figure S1.* Mean cycle lengths across cycles (horizontal axis) demonstrating that data for the first and final cycles were significantly lower than all other cycles.

| Table S2 *Predictors of Cycle Variability (Model 2)* | | | | |  |
| --- | --- | --- | --- | --- | --- |
| **Parameter** | ***β* (*SE*)** | ***t*** | ***p*** | ***R*^2^** | |
| *Coefficient of Variation* |  |  |  | .01 | |
| Age | .05 (.01)* | 4.25 | < .001 |  | |
| Chronic Illness | .02 (.01)* | 2.30 | .02 |  | |
| Relationship Status | -.01 (.01) | -.74 | .46 |  | |
| Smoking | .07 (.01)*** | 6.64 | < .001 |  | |
| Indian vs. White | .01 (.01) | 1.13 | .26 |  | |
| Asian vs. White | -.01 (.01) | .1.21 | .23 |  | |
| Hispanic vs. White | -.02 (.01) | -1.62 | .11 |  | |
| Black vs. White | -.01 (.01) | -1.20 | .23 |  | |
| Weight | -.001 (.01) | -.09 | .93 |  | |
| *Note. R*^2^ refers to variance explained by all effects in model. **p* < .05, ***p* < .01, ****p* < .001. | | | | |  |

| Table S3 *Autoregressive and Cross-Lagged Predictors of Cycle Length (Model 3)* | | | | |
| --- | --- | --- | --- | --- |
| **Parameter** | ***β* (*SE*)** | ***t*** | ***p*** | ***R*^2^** |
| *Random Intercept (Between-Portion of Model)* |  |  |  | .07 |
| Age | -.22 (.01)*** | -16.62 | < .001 |  |
| Chronic Illness | -.02 (.01)* | -2.00 | .046 |  |
| Relationship Status | .04 (.01)*** | 3.26 | .001 |  |
| Smoking | -.04 (.01)*** | -3.57 | < .001 |  |
| Indian | .002 (.01) | .15 | .88 |  |
| Asian | .07 (.01)*** | 6.01 | < .001 |  |
| Hispanic | .08 (.01)*** | 6.37 | < .001 |  |
| Black | -.06 (.01)*** | -4.85 | < .001 |  |
| Weight | .03 (.01)** | 1.64 | .008 |  |
| *Autoregressive Paths (Within-Portion of Model)* |  |  |  |  |
| Cycle Length 1 → Cycle Length 2 | -.04 (.01)*** | -3.52 | < .001 | .002 |
| Cycle Length 2 → Cycle Length 3 | -.08 (.01)*** | -6.84 | < .001 | .01 |
| Cycle Length 3 → Cycle Length 4 | -.10 (.01)*** | -8.46 | < .001 | .01 |
| Cycle Length 4 → Cycle Length 5 | -.08 (.01)*** | -6.53 | < .001 | .01 |
| Cycle Length 5 → Cycle Length 6 | -.07 (.01)*** | -5.99 | < .001 | .01 |
| Cycle Length 6 → Cycle Length 7 | -.07 (.01)*** | -5.94 | < .001 | .01 |
| Cycle Length 7 → Cycle Length 8 | -.07 (.01)*** | -5.91 | < .001 | .01 |
| Cycle Length 8 → Cycle Length 9 | -.06 (.01)*** | -4.93 | < .001 | .004 |
| Cycle Length 9 → Cycle Length 10 | -.07 (.01)*** | -5.90 | < .001 | .01 |
| *Effects of Sexual Motivation (Within-Portion of Model)* |  |  |  |  |
| Sexual Motivation 1 → Cycle Length 2 | .004 (.01) | .37 | .71 |  |
| Sexual Motivation 2 → Cycle Length 3 | -.01 (.01) | -.64 | .53 |  |
| Sexual Motivation 3 → Cycle Length 4 | .001 (.01) | .06 | .95 |  |
| Sexual Motivation 4 → Cycle Length 5 | .01 (.01) | .77 | .44 |  |
| Sexual Motivation 5 → Cycle Length 6 | -.02 (.01) | -1.44 | .15 |  |
| Sexual Motivation 6 → Cycle Length 7 | -.01 (.01) | -.88 | .38 |  |
| Sexual Motivation 7 → Cycle Length 8 | .01 (.01) | .98 | .33 |  |
| Sexual Motivation 8 → Cycle Length 9 | -.01 (.01) | -.46 | .65 |  |
| Sexual Motivation 9 → Cycle Length 10 | .02 (.01) | .1.21 | .23 |  |
| *Note. R*^2^ refers to variance explained by all effects in model. **p* < .05, ***p* < .01, ****p* < .001. | | | | |

| Table S4  *Autoregressive and Cross-Lagged Predictors of Sexual Motivation (Model 3)* | | | | |
| --- | --- | --- | --- | --- |
| **Parameter** | ***β* (*SE*)** | ***t*** | ***p*** | ***R*^2^** |
| *Random Intercept (Between-Portion of Model)* |  |  |  | .03 |
| Age | -.08 (.01)*** | -6.71 | < .001 |  |
| Chronic Illness | .01 (.01) | 1.08 | .28 |  |
| Relationship Status | .17 (.01)*** | 14.79 | < .001 |  |
| Smoking | .08 (.01)*** | 7.36 | < .001 |  |
| Indian | .02 (.01)* | 2.06 | .04 |  |
| Asian | .02 (.01) | 1.39 | .17 |  |
| Hispanic | -.003 (.01) | -.32 | .75 |  |
| Black | .01 (.01) | 1.27 | .20 |  |
| Weight | .03 (.01)** | 2.81 | .005 |  |
| Age |  |  |  |  |
| *Autoregressive Paths (Within-Portion of Model)* |  |  |  |  |
| Sexual Motivation 1 → Sexual Motivation 2 | .59 (.01)*** | 82.60 | < .001 | .35 |
| Sexual Motivation 2 → Sexual Motivation 3 | .57 (.01)*** | 73.15 | < .001 | .32 |
| Sexual Motivation 3 → Sexual Motivation 4 | .50 (.01)*** | 58.58 | < .001 | .25 |
| Sexual Motivation 4 → Sexual Motivation 5 | .35 (.01)*** | 32.17 | < .001 | .12 |
| Sexual Motivation 5 → Sexual Motivation 6 | .25 (.01)*** | 19.94 | < .001 | .06 |
| Sexual Motivation 6 → Sexual Motivation 7 | .17 (.01)*** | 12.51 | < .001 | .03 |
| Sexual Motivation 7 → Sexual Motivation 8 | .16 (.01)*** | 11.15 | < .001 | .03 |
| Sexual Motivation 8 → Sexual Motivation 9 | .26 (.01)*** | 19.59 | < .001 | .07 |
| Sexual Motivation 9 → Sexual Motivation 10 | .34 (.01)*** | 29.63 | < .001 | .12 |
| *Effects of Cycle Length (Within-Portion of Model)* |  |  |  |  |
| Cycle Length 1 → Sexual Motivation 2 | -.09 (.01)*** | -9.46 | < .001 |  |
| Cycle Length 2 → Sexual Motivation 3 | -.10 (.01)*** | -11.24 | < .001 |  |
| Cycle Length 3 → Sexual Motivation 4 | -.10 (.01)*** | -9.65 | < .001 |  |
| Cycle Length 4 → Sexual Motivation 5 | -.08 (.01)*** | -7.27 | < .001 |  |
| Cycle Length 5 → Sexual Motivation 6 | -.07 (.01)*** | -5.93 | < .001 |  |
| Cycle Length 6 → Sexual Motivation 7 | -.07 (.01)*** | -5.19 | < .001 |  |
| Cycle Length 7 → Sexual Motivation 8 | -.06 (.01)*** | -4.84 | < .001 |  |
| Cycle Length 8 → Sexual Motivation 9 | -.08 (.01)*** | -6.31 | < .001 |  |
| Cycle Length 9 → Sexual Motivation 10 | -.09 (.01)*** | -7.79 | < .001 |  |
| *Note. R*^2^ refers to variance explained by all effects in model. **p* < .05, ***p* < .01, ****p* < .001. | | | | |

| Table S5  *Autoregressive and Cross-Lagged Predictors of Physical Complaints (Model 3)* | | | |  |
| --- | --- | --- | --- | --- |
| **Parameter** | ***β* (*SE*)** | ***t*** | ***p*** | ***R*^2^** |
| *Random Intercept (Between-Portion of Model)* |  |  |  | .02 |
| Age | -.03 (.01)* | -2.18 | .03 |  |
| Chronic Illness | .11 (.01)*** | 10.01 | < .001 |  |
| Relationship Status | -.03 (.01)* | -2.19 | .03 |  |
| Smoking | .01 (.01) | 1.30 | .19 |  |
| Indian | .02 (.01) | 1.46 | .15 |  |
| Asian | -.02 (.01) | -1.92 | .06 |  |
| Hispanic | -.05 (.01)*** | -4.54 | < .001 |  |
| Black | -.06 (.01)*** | -5.41 | <.001 |  |
| Weight |  |  |  |  |
| *Autoregressive Paths (Within-Portion of Model)* |  |  |  |  |
| Physical Complaints 1 → Physical Complaints 2 | .47 (.01)*** | 50.07 | < .001 | .22 |
| Physical Complaints 2 → Physical Complaints 3 | .40 (.01)*** | 37.94 | < .001 | 16 |
| Physical Complaints 3 → Physical Complaints 4 | .27 (.01)*** | 21.77 | < .001 | .07 |
| Physical Complaints 4 → Physical Complaints 5 | .23 (.01)*** | 18.92 | < .001 | .05 |
| Physical Complaints 5 → Physical Complaints 6 | .20 (.01)*** | 16.34 | < .001 | .04 |
| Physical Complaints 6 → Physical Complaints 7 | .29 (.01)*** | 24.42 | < .001 | .08 |
| Physical Complaints 7 → Physical Complaints 8 | .29 (.01)*** | 25.01 | < .001 | .08 |
| Physical Complaints 8 → Physical Complaints 9 | .30 (.01)*** | 26.66 | < .001 | .09 |
| Physical Complaints 9 → Physical Complaints 10 | .44 (.01)*** | 45.59 | < .001 | .19 |
| *Effects of Cycle Length (Within-Portion of Model)* |  |  |  |  |
| Cycle Length 1 → Physical Complaints 2 | -.05 (.01)*** | -5.19 | < .001 |  |
| Cycle Length 2 → Physical Complaints 3 | -.06 (.01)*** | -5.87 | < .001 |  |
| Cycle Length 3 → Physical Complaints 4 | -.07 (.01)*** | -6.29 | < .001 |  |
| Cycle Length 4 → Physical Complaints 5 | -.05 (.01)*** | -4.25 | < .001 |  |
| Cycle Length 5 → Physical Complaints 6 | -.05 (.01)*** | -4.11 | < .001 |  |
| Cycle Length 6 → Physical Complaints 7 | -.07 (.01)*** | -6.02 | < .001 |  |
| Cycle Length 7 → Physical Complaints 8 | -.08 (.01)*** | -6.95 | < .001 |  |
| Cycle Length 8 → Physical Complaints 9 | -.07 (.01)*** | -5.79 | < .001 |  |
| Cycle Length 9 → Physical Complaints 10 | -.07 (.01)*** | -6.28 | < .001 |  |
| *Effects of Physical Complaints (Within-Portion of Model)* |  |  |  |  |
| Physical Complaints 1 → Cycle Length 2 | .01 (.01) | .74 | .46 |  |
| Physical Complaints 2 → Cycle Length 3 | .02 (.01) | 1.66 | .10 |  |
| Physical Complaints 3 → Cycle Length 4 | .02 (.01) | 1.35 | .18 |  |
| Physical Complaints 4 → Cycle Length 5 | .03 (.01)* | 1.99 | .047 |  |
| Physical Complaints 5 → Cycle Length 6 | .01 (.01) | .59 | .55 |  |
| Physical Complaints 6 → Cycle Length 7 | .01 (.01) | .49 | .63 |  |
| Physical Complaints 7 → Cycle Length 8 | .01 (.01) | .51 | .61 |  |
| Physical Complaints 8 → Cycle Length 9 | .02 (.01) | 1.22 | .22 |  |
| Physical Complaints 9 → Cycle Length 10 | .01 (.01) | 1.07 | .30 |  |
| *Note. R*^2^ refers to variance explained by all effects in model. **p* < .05, ***p* < .01, ****p* < .001. | | | | |

Table S6

*Results of Sensitivity Analyses*

| *Autoregressive Effects* | **Original Model** | **Logged on ≥25% of days** | **Logged on ≥50% of days** | **Logged on ≥75% of days** | **Logged on 100% of days** |
| --- | --- | --- | --- | --- | --- |
| **Cycle Length 🡪 Cycle Length** | -0.08*** | -0.08*** | -0.08*** | -0.08*** | -0.08*** |
| **Sexual Motivation 🡪 Sexual Motivation** | 0.40*** | 0.47*** | 0.51*** | 0.56*** | 0.48*** |
| *Cross-lagged Effects* | **Original Model** | **Logged on ≥25% of days** | **Logged on ≥50% of days** | **Logged on ≥75% of days** | **Logged on 100% of days** |
| **Cycle Length 🡪 Sexual Motivation** | -0.10*** | -0.15*** | -0.18*** | -0.19*** | -0.18*** |
| **Sexual Motivation 🡪 Cycle Length** | -0.001 | 0.008 | 0.007 | 0.02 | 0.02 |

*Note.* Shown here are standardized coefficients and statistical significance for omnibus tests of key effects across sensitivity analyses. ****p* < 0.001.
